# Sensitive Dentine

**Published:** 1888-12

**Authors:** 


### SELECTIONS.

### Sensitive Dentine.

One of the difficulties to be overcome in tooth filling is the occasional extreme sensitiveness of the dentine; and it requires considerable courage on the part of the patient to submit to the necessary cutting. Healthy dentine is endowed with but little sensibility, for if a tooth be accidentally chipped so as to expose a portion of the dentine without opening the pulp chamber, it can at first be touched without giving rise to pain, but after twenty-four hours' exposure to the fluids of the mouth it becomes irritable. Hyperæsthesia of the dental fibrils may follow any form of exposure—that due to caries, erosion, or fracture; and when present it varies considerably in intensity, not only in different teeth, but in different parts of the same tooth. Immediately under the enamel, in proximity to the pulp, especially the fibres radiating from the cornua of and just beneath the appreciably softened carious bone, are the points of greatest sensibility. The former two are explained by anatomical facts. The dentinal fibres end at the periphery by forming a dense network, and open into the so-called inter-globular spaces, which, however, are filled with protoplasm similar to that contained in the lacunæ of bone, the whole forming the granular layer of Tomes, and near the pulp the fibrils are both more numerous and of larger calibre. The deepest portion of the diseased bone is probably most sensitive, because here the calcareous material has been removed, exposing the fibrils, while it has not gone far enough to destroy them. It will readily be understood from this that superficial cavities are often very sensitive; then, as the disease progresses and this portion of the dentine is destroyed, there comes a time when there is but little pain from contact with acrid fluid or solid substances, but acute sensitiveness is again met with as the pulp is approached. Other things being equal, soft teeth, from their relative greater quantity of organic matter, will be more sensitive than hard teeth, although there are many exceptions to this rule; also the teeth of the young more than those of the aged. The various methods of treatment may be summed up under the following heads: (*a*) operative measures, (*b*) desiccation, (*c*) cauterization, (*d*) local anæsthesia; and this is probably the order of their efficiency, although combinations are often valuable. (*a*) Sharp instruments, rapid motion of the engine, and taking advantage of anatomical knowledge by cutting away from the pulp and its cornua and across the line of the fibrils, will suffice in most cases. After the insertion of a temporary plastic filling for a few months, it will generally be found that most of the sensitiveness has disappeared; and this is the best of all treatment if the patient is under constant supervision. (*b*) In order to get thorough dryness, the rubber dam must be adjusted and the cavity swabbed out repeatedly with cotton wool dipped in $CHCI_3$ or absolute alcohol, or, better still, a current of hot air passed through the cavity. Chloride of zinc acts partly as a desiccator and partly as an escharotic, but its application is usually very painful, and its use contra-indicated when the pulp is near. (*c*) Carbolic acid, chloride of zinc, nitrate of silver, and caustic soda all have their advocates—carbolic acid, perhaps, being the most general favorite. Arsenious acid left in for a few hours is most efficacious; but, owing to the numerous cases of death of the pulp resulting, is now rarely resorted to. (*d*) Cocaine alone, or in conjunction with sulphuric ether, although of great use as a local anæsthetic in treating pulps, has not proved of much service for sensitive dentine, as it is not readily absorbed. Other drugs, such as menthol and aconite, are equally disappointing. Dr. Ottolengui's method seems, as far as experience has gone, to give good results.

He first dries the cavity with hot air, and sometimes uses carbolic acid as an escharotic, and then anæsthetises the dentine with ether spray. It is maintained that the pain produced is far less than the operation of cutting by any other method. It may be interesting to note the statement that, where teeth are forcibly wedged apart for the purpose of gaining room for filling, the pain of excavating is much diminished, presumably from constriction of the nerve as it enters the apical foramen. Cocaine injection into the gum, as used for extractions, has also been recommended, but has not given encouraging results.—*The Lancet*, October 27, 1888.

